# Easy Test for Arsenic in Fabrics

**Published:** 1880-12

**Authors:** 


### EASY TEST FOR ARSENIC IN FABRICS.

The following plan is recommended by Dr. Henry Barnes, in the *Practitioner*, as an easy plan to detect arsenic in paper hangings or in other suspected fabric:

Immerse the suspected paper in strong ammonia, on a white plate or saucer; if the ammonia becomes blue, the presence of a salt of copper is proved; then drop a crystal of nitrate of silver into the blue liquid, and if any arsenic be present, the crystal will become coated with yellow arseniate of silver, which will disappear on stirring.

This test is not so well known as it should be. It is mentioned in some of the text-books, but we are not aware to whom the credit of its first introduction is due.

